# Supplementary material for: Room-temperature X-ray fragment screening with serial crystallography
Source: Nat Commun. 2025 Oct 13;16:9089. doi: 10.1038/s41467-025-64918-6 (PMC12518807; doi:10.1038/s41467-025-64918-6)
Supplement: Supplementary file 1 — Supplementary Information [file 41467_2025_64918_MOESM1_ESM.pdf]

Supplementary information

Room-temperature X-ray fragment screening with serial crystallography

Sebastian Günther[1,*], Pontus Fischer[1], Marina Galchenkova[1], Sven Falke[1], Patrick Y.A. Reinke[1], Sreevidya Thekku Veedu[1], Ana Carolina Rodrigues[1], Johanna Senst[1], Lars Gumprecht[1], Jan Meyer[1], Henry N. Chapman[1,2,3], Miriam Barthelmess[1], and Alke Meents[1,*].

1. Center for Free-Electron Laser Science CFEL, Deutsches Elektronen-Synchrotron DESY, Notkestr. 85, 22607 Hamburg, Germany
2. The Hamburg Centre for Ultrafast Imaging, Luruper Chaussee 149, 22761 Hamburg, Germany
3. Department of Physics, University of Hamburg, Luruper Chaussee 149, 22761 Hamburg, Germany

* Corresponding authors:
Sebastian Günther, sebastian.guenther@desy.de
Alke Meents, alke.meents@desy.de

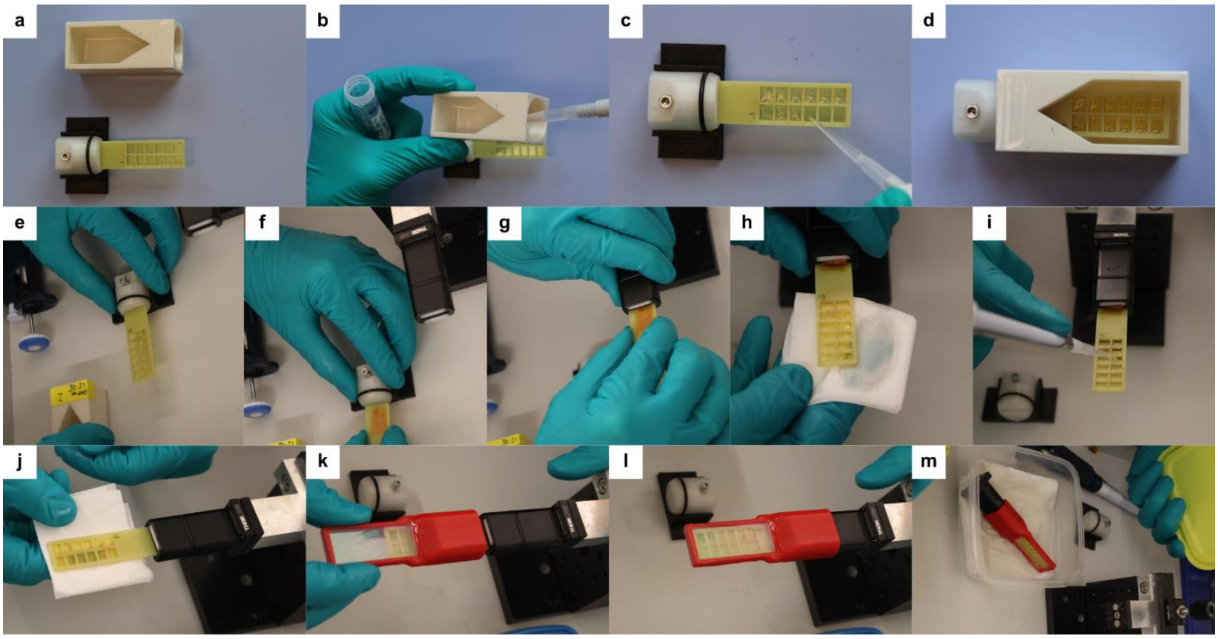

Supplementary Figure 1:
Sample preparation for fixed-target serial crystallography experiment. Crystals are directly grown on the sample holder that is equipped with 12 compartments and a microporous polyimide membrane using 3D-printed crystallization chambers and holders (**a**). Crystallization solution is pipetted into the chamber (**b**). Afterwards a protein/crystallization solution mix is added to each compartment (**c**). The sample holder is inserted into the crystallization chamber (**d**) and placed in an incubator. For fragment soaking after crystal growth, the sample holder is taken out of the chamber (**e**), removed from the 3D-printed holder (**f**) and inserted into a kinematic mount, which will later also be used for data collection (**g**). Crystallization solution is blotted through the pores (**h**) and solutions containing the fragments are added to the crystals by pipetting (one fragment per compartment) (**i**). Afterwards the sample holder is transferred back into the crystallization chamber and placed in an incubator for extended soaking times. For data collection, the sample holder is again inserted into the kinematic mount and the soaking solution is removed by blotting (j). A protective sleeve with Mylar windows is slid over the sample (**k,l**). For transport to the goniometer the sample holder is transferred into a box with wet tissues to prevent dehydration (**m**). Steps **e** to **m** are conducted in a glove box to ensure high relative humidity levels.

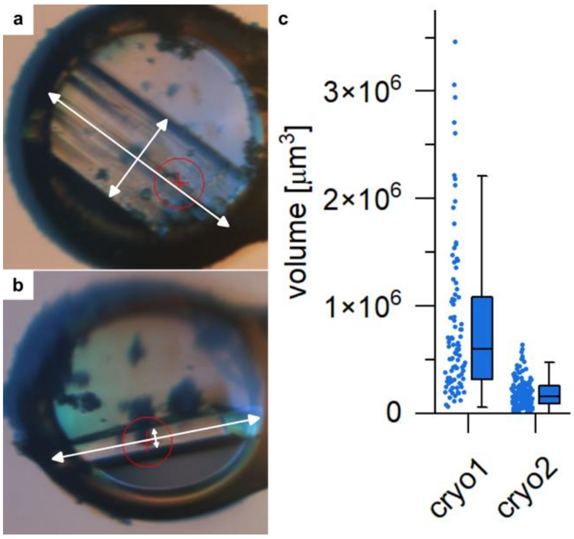

Supplementary Figure 2:

Sizes of crystals used for screen cryo1 and cryo2 differ. **a** and **b**, Images for two crystals from data collection with measured sizes indicated by white arrows. The red circle represents the X-ray beam (50 µm diameter). **c**, the non-visible third dimension of the crystals was estimated to be 1/3 of the shorter dimension and used to estimate crystal volume. For cryo1 94 images and for cryo2 155 images could be analyzed. For each screen, individual datapoints (left) are depicted next to summarizing box plots (right) that depict the lower limit of the second quartile range (minimum), the median (center) and the upper limit of the third quartile (maximum). Whiskers represent 1.5-fold the interquartile range.

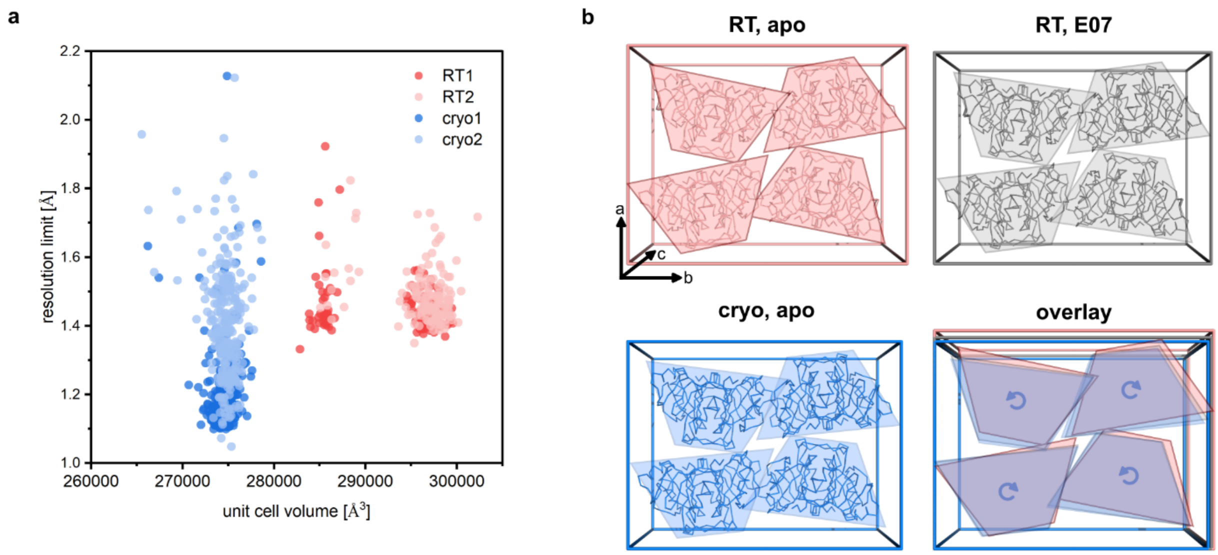

Supplementary Figure 3:

Data collection at room-temperature and cryogenic temperatures leads to variations in the unit cell and subsequently also in the structure of FosAKP. **a**, both unit cell clusters for the RT datasets show a similar resolution distribution. **b**, unit cells with molecules indicating the packing within the crystal for each of the representative structures highlighted in Fig. 3. The overlay of the three unit-cells demonstrates the rotation and shifting of each molecule within the unit cell in the different clusters.

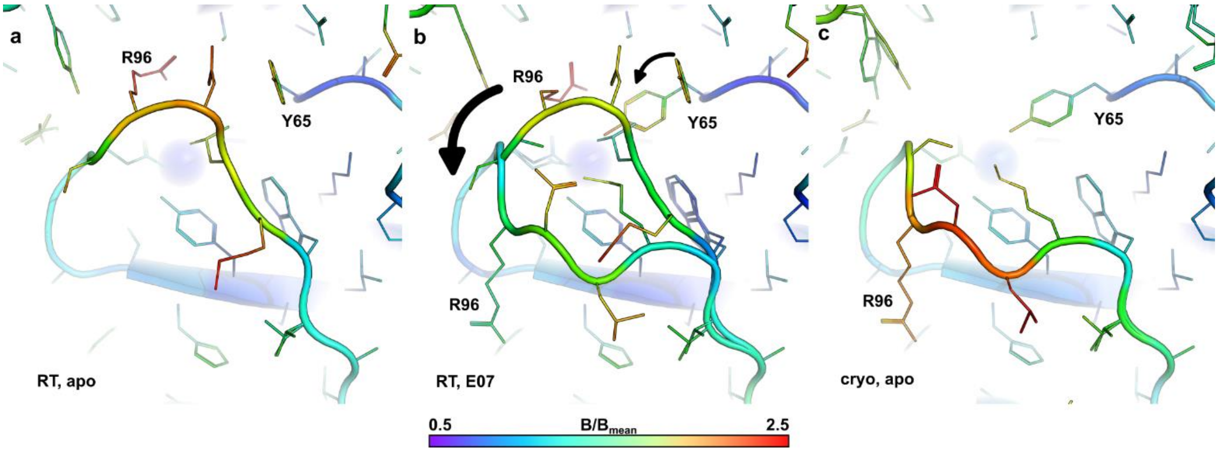

Supplementary Figure 4:

High flexibility of active-site loop of FosAKP. The residues of the K^{+}-binding loop of active site I exhibits high B values in all observed conformations (closed state in RT apo, **a**; double conformation in RT E07 fragment structure, **b**; more open state in cryo apo, **c**). For enhanced comparability of RT and cryo structures, all B values were normalized to the structure's mean B for all protein atoms. The structure is colored by the normalized B value according to the depicted scale.

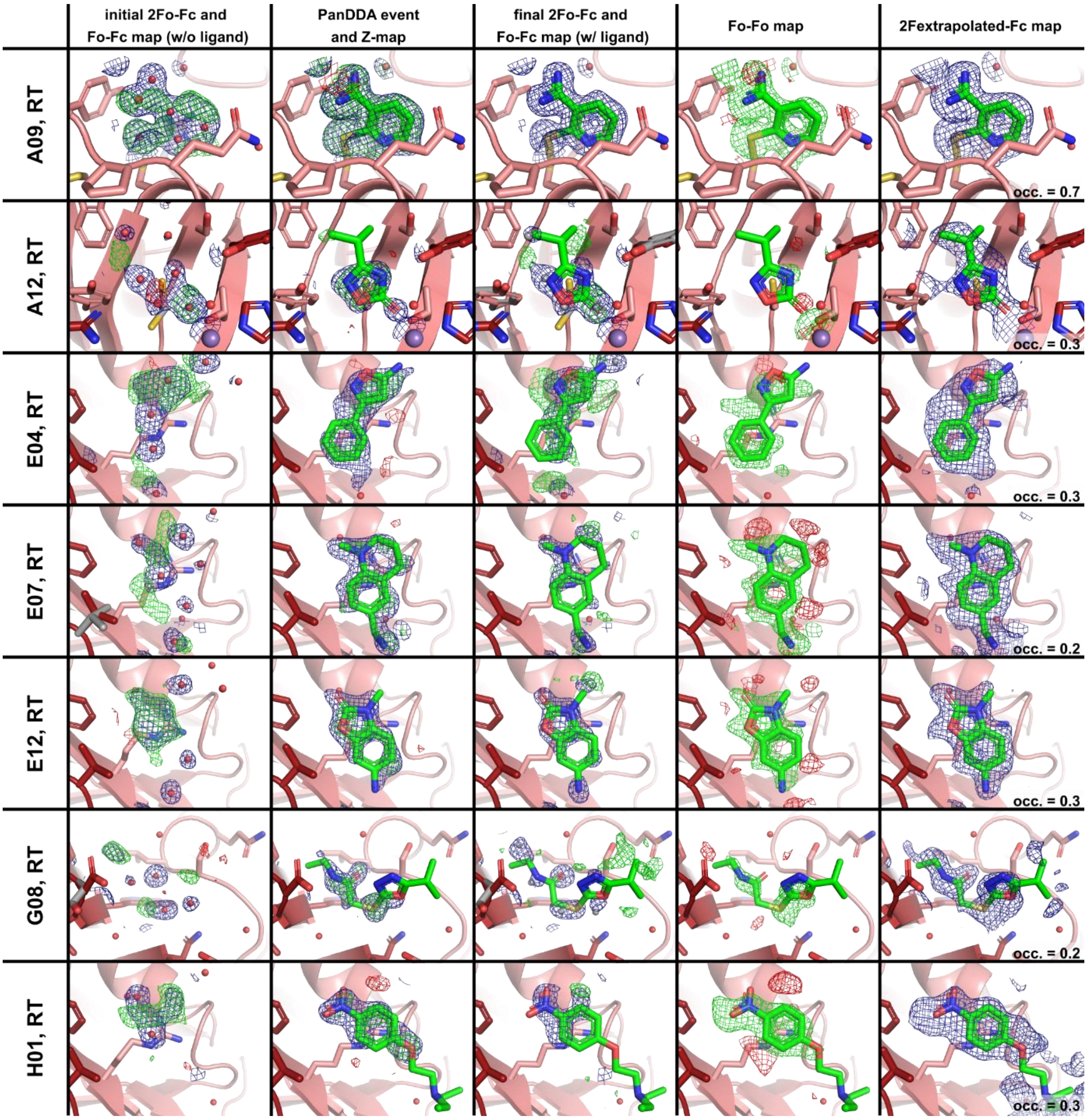

Supplementary Figure 5:

PanDDA and Xtrapol8 enable identification of bound fragments. All seven unique binding events observed over the two RT screens are shown with the 2Fo-Fc (blue mesh, at 1 σ) and Fo-Fc map (green/red mesh, at 3 σ) from the initial automatic refinement before the addition of any fragment model (first column) and of the fully refine structure with modeled fragments (third column). PanDDA event maps (blue mesh, at 2 σ) and Z-map (green/red mesh, at 3 σ) are shown (second column). Isomorphous difference maps (Fo-Fo) and maps using extrapolated structure factors (2Fextrapolated-Fc) are often used in serial crystallography to detect low-occupancy states. Q-weighted Fo,ligand-Fo,groundstate maps are shown in the fourth column (green/red mesh, at 3 σ). The fifth column shows maps calculated with extrapolated structure factors as derived from Xtrapol8. Assumed occupancy of the ligand-bound state is indicated (blue mesh, 1 σ). Maps are carved at 2 Å around the fragments. Water molecules within 3 Å of the fragment and protein residues within 4 Å are shown.

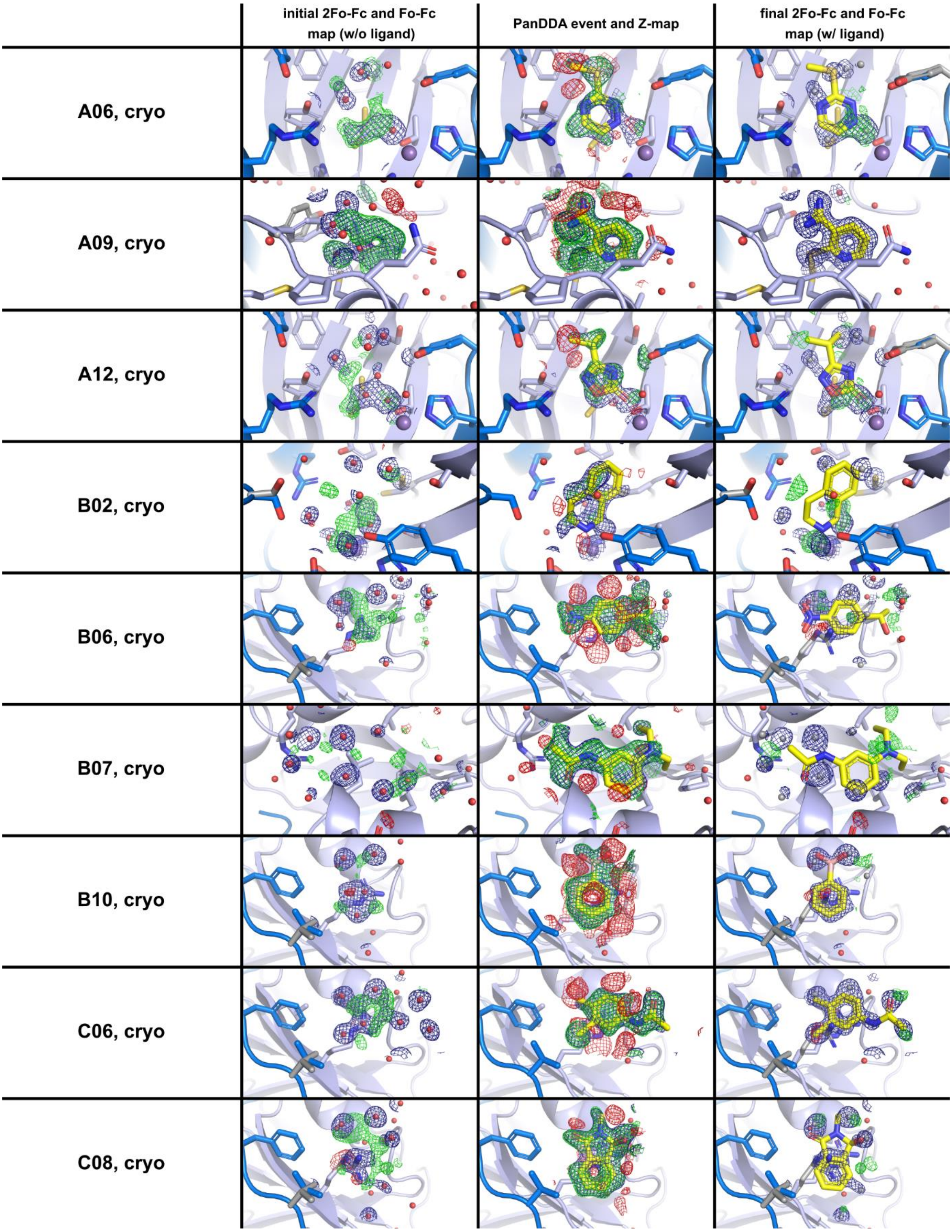
initial 2Fo-Fc and Fo-Fc map (w/o ligand)
PanDDA event and Z-map
final 2Fo-Fc and Fo-Fc map (w/ ligand)
A06, cryo
A09, cryo
A12, cryo
B02, cryo
B06, cryo
B07, cryo
B10, cryo
C06, cryo
C08, cryo

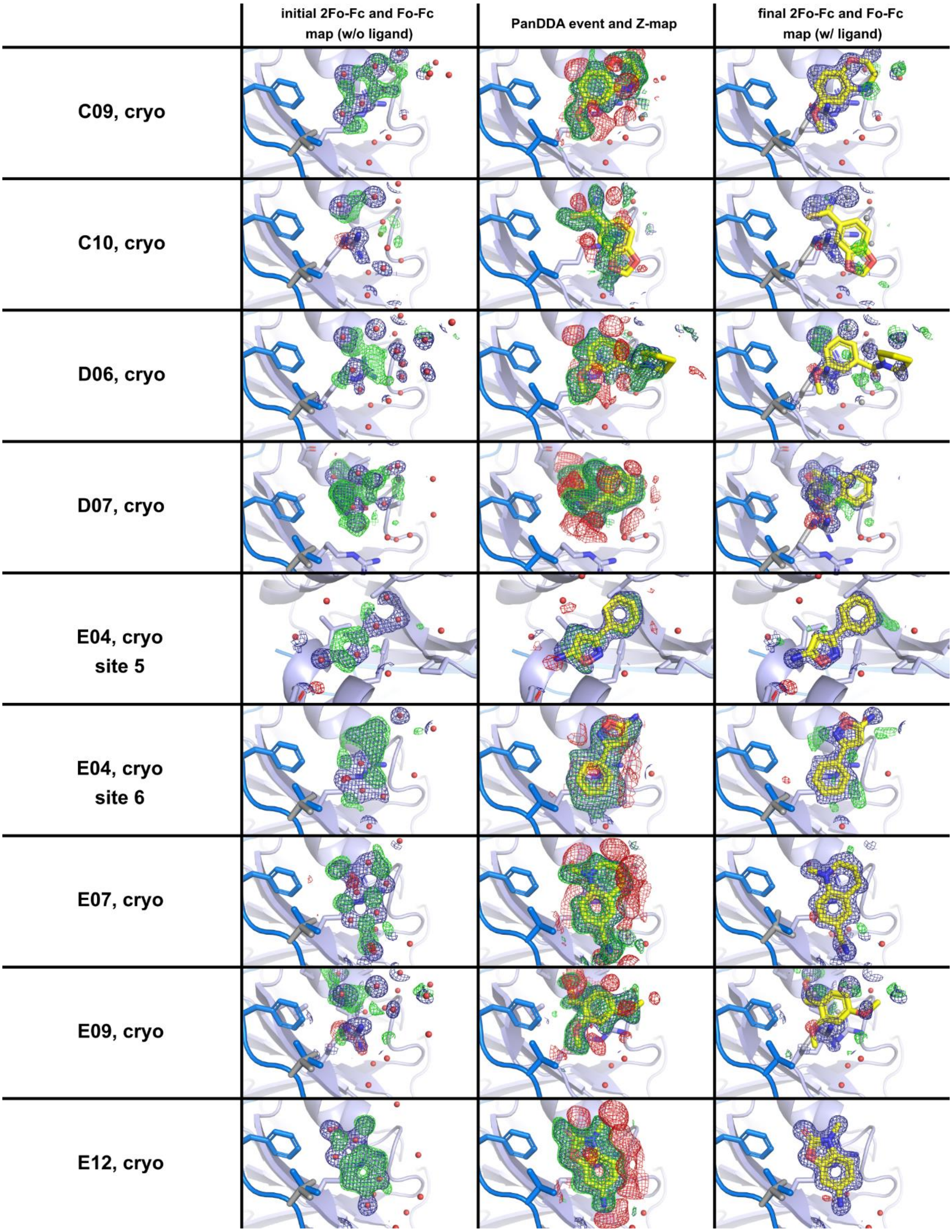
initial 2Fo-Fc and Fo-Fc map (w/o ligand)
PanDDA event and Z-map
final 2Fo-Fc and Fo-Fc map (w/ ligand)
C09, cryo
C10, cryo
D06, cryo
D07, cryo
E04, cryo site 5
E04, cryo site 6
E07, cryo
E09, cryo
E12, cryo

	initial 2Fo-Fc and Fo-Fc map (w/o ligand)	PanDDA event and Z-map	final 2Fo-Fc and Fo-Fc map (w/ ligand)
**F02, cryo site 5**	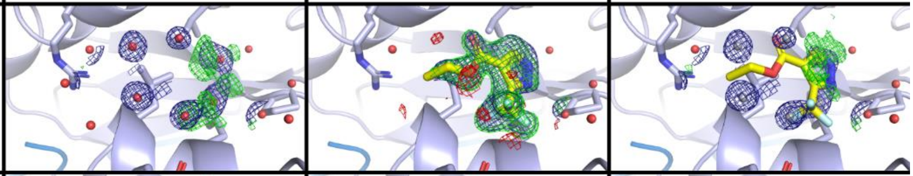		
**F02, cryo site 6**	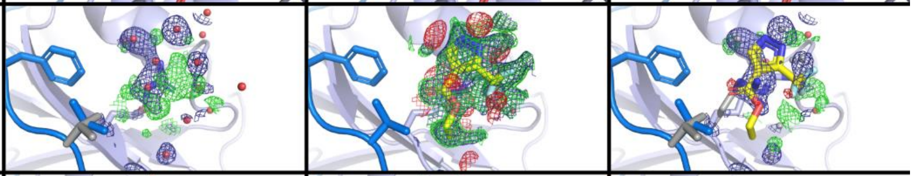		
**F04, cryo**	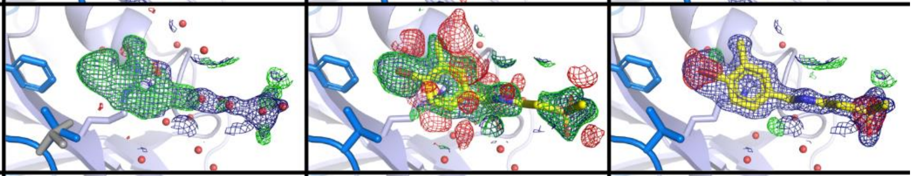		
**F08, cryo**	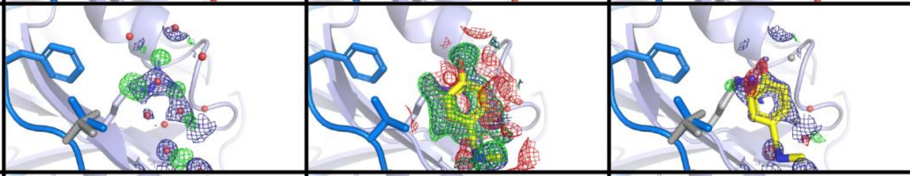		
**F09, cryo**	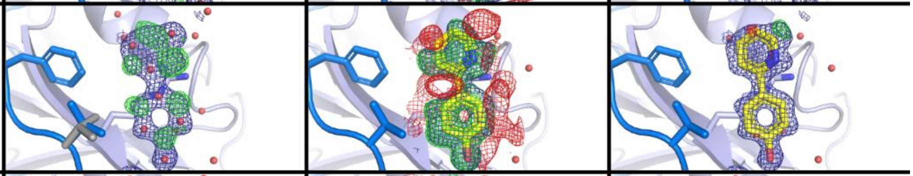		
**F10, cryo**	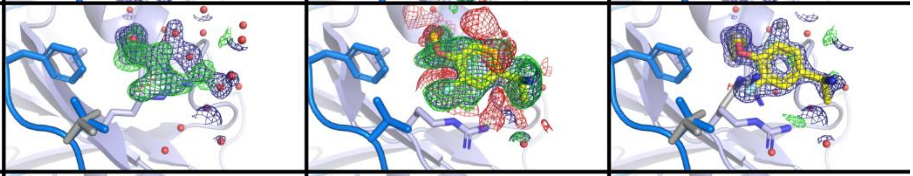		
**G02, cryo**	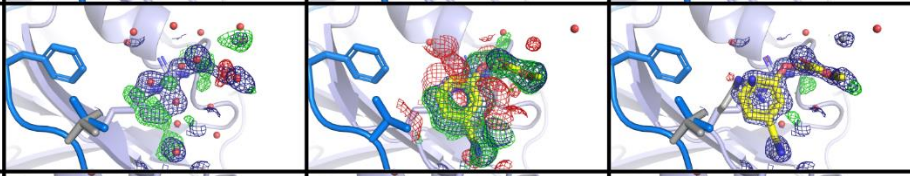		
**G03, cryo site 5**	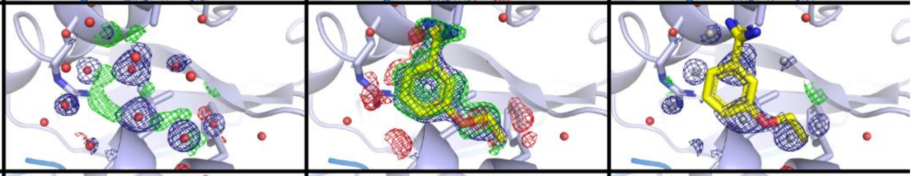		
**G03, cryo site 6**	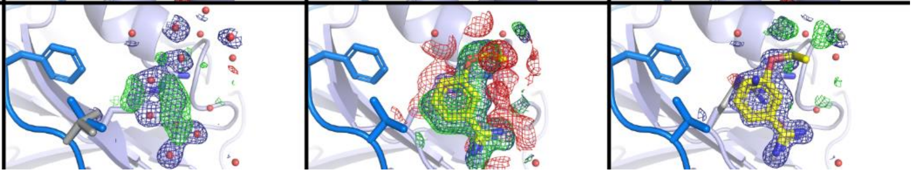		

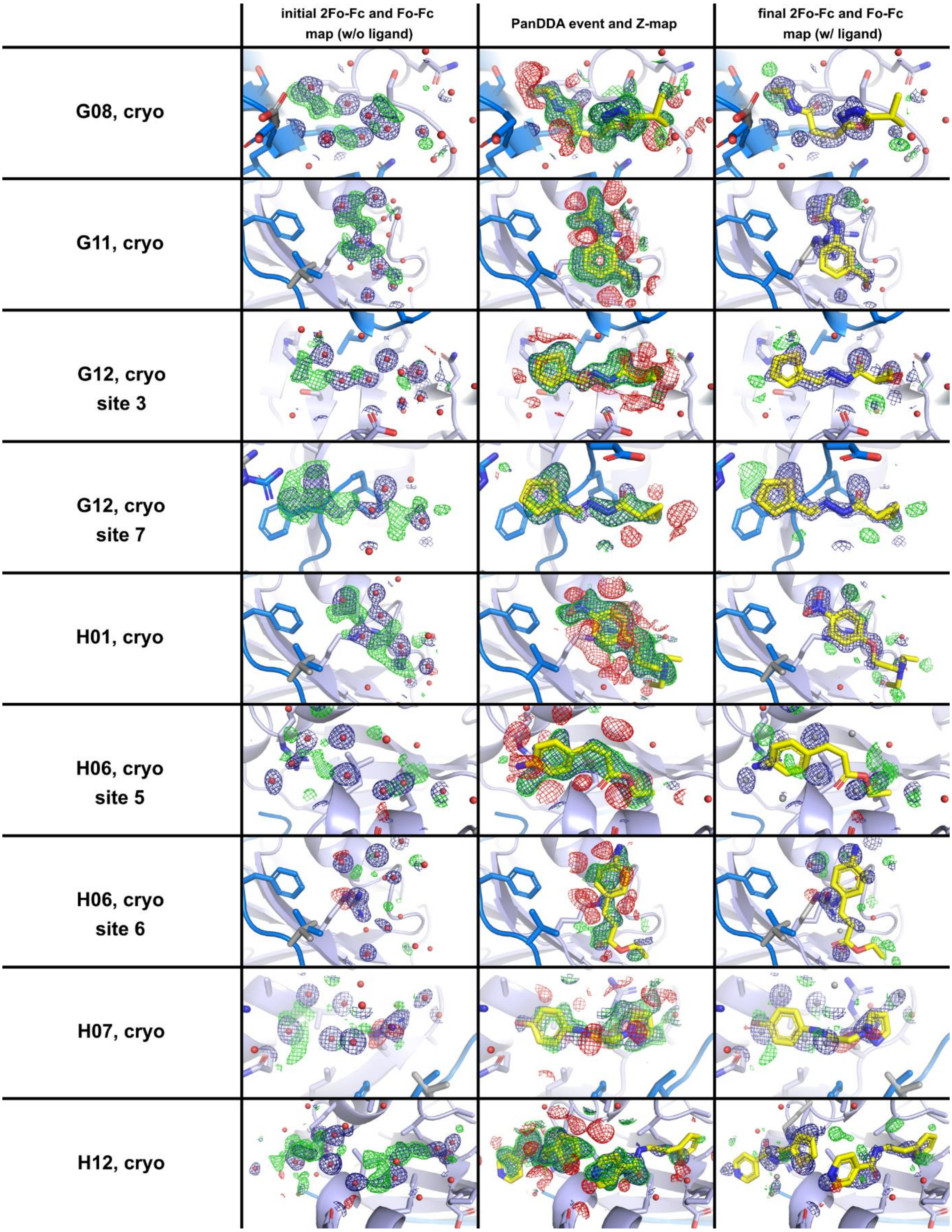

Supplementary Figure 6:

PanDDA enables identification of bound fragments. All 31 unique binding events observed over the 2 cryo screens are shown with the 2Fo-Fc (blue mesh, at 1 σ) and Fo-Fc map (green/red mesh, at 3 σ) from the initial automatic refinement before the addition of any fragment model (left column) and of the fully refine structure with modeled fragments (right column). PanDDA event maps (blue mesh, at 2 σ) and Z-map (green/red mesh, at 3 σ) are shown (middle column). Maps are carved at 2 Å

around the fragments. Water molecules within 3 Å of the fragment and protein residues within 4 Å are shown. Water molecules colored grey in the final model (right column) are in alternate conformation B, while the fragment is modeled in conformation A.

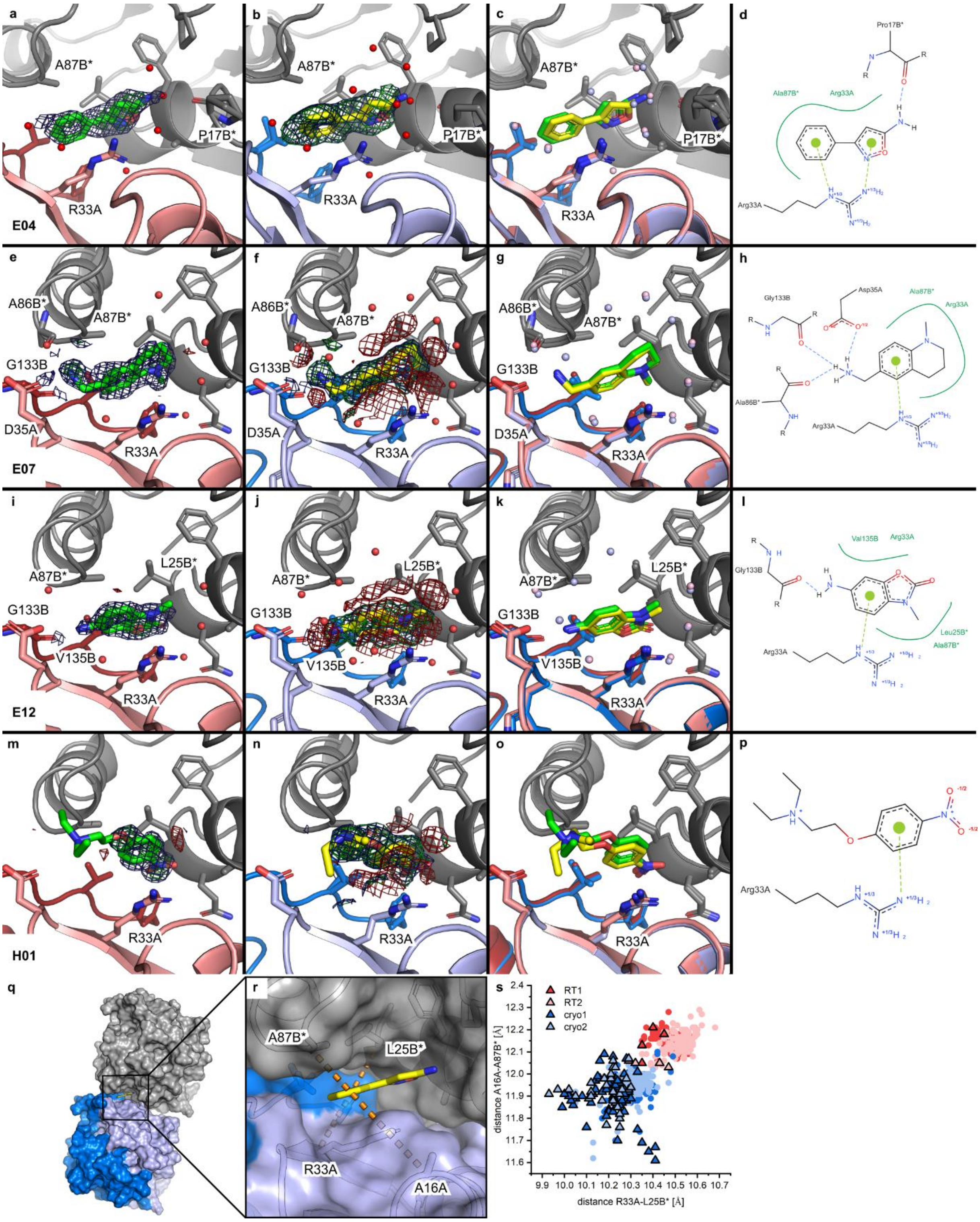

Supplementary Figure 7:

Fragments identified in the RT and cryo screens binding at site 6. Refined RT structures (panels **a**, **e**, **I**, **m**) and cryo structures (panels **b**, **f**, **j**, **n**) are shown with PanDDA event map (blue mesh, 2 σ level) and Z-map (green mesh, 3 σ level) drawn around the ligand (carved at 2 Å). The second FosAKP homodimer that forms the crystal contact is shown in grey. Overlay of structures from both temperatures is shown in panels **c**, **g**, **k**, **o** and interaction diagrams of ligands with the protein are shown in panels **d**, **h**, **l**, **p**. **q**, crystal contact that form binding site 6. **r**, closeup view of site 6 for

cryo structure with ligand E04. The two residue pairs that were used to measure the width of the pocket are indicated. **s**, analysis of distance for the residue pair in **r** for all automatically refined structures (round symbols). Datasets with ligands identified at site 6 are depicted as triangle.

a

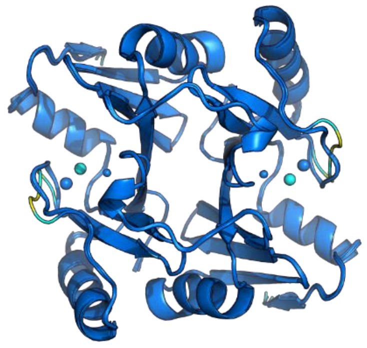

b

	apo_RT	apo_cryo	apo_cryo_PDB	FCN_cryo_PDB	AF3_model1	AF3_model2	AF3_model3	AF3_model4	AF3_model5
apo_RT	-	-	-	-	-	-	-	-	-
apo_cryo	0.561	-	-	-	-	-	-	-	-
apo_cryo_PDB	0.564	0.314	-	-	-	-	-	-	-
FCN_cryo_PDB	0.667	0.516	0.49	-	-	-	-	-	-
AF3_model1	0.642	0.48	0.463	0.252	-	-	-	-	-
AF3_model2	0.65	0.479	0.476	0.297	0.16	-	-	-	-
AF3_model3	0.647	0.494	0.476	0.248	0.085	0.169	-	-	-
AF3_model4	0.641	0.495	0.488	0.262	0.135	0.136	0.143	-	-
AF3_model5	0.696	0.522	0.535	0.488	0.401	0.37	0.404	0.389	-

c

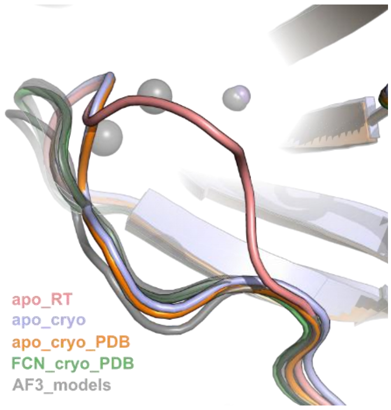

Supplementary Figure 8:

FosAKP RT K^+-loop conformation is not predicted by AlphaFold3 (AF3). **a**, Overall the structure is predicted with very high confidence. The top five results for structures of the FosAKP homodimer with Mn^{2+} and K^+ ions show a slight variability in the K^+-binding loop conformation. Coloring follows pLDDT values (blue, 90-100; confident, 70-90; low, 50-70). **b**, pairwise RMSD values for apo structures from this study, previously published structures of the apo form (PDB 5V91) and with bound Fosfomycin (PDB 5V3D) and the AF3 models. **c**, conformation of K^+-binding loop for the 9 different structures listed in b.

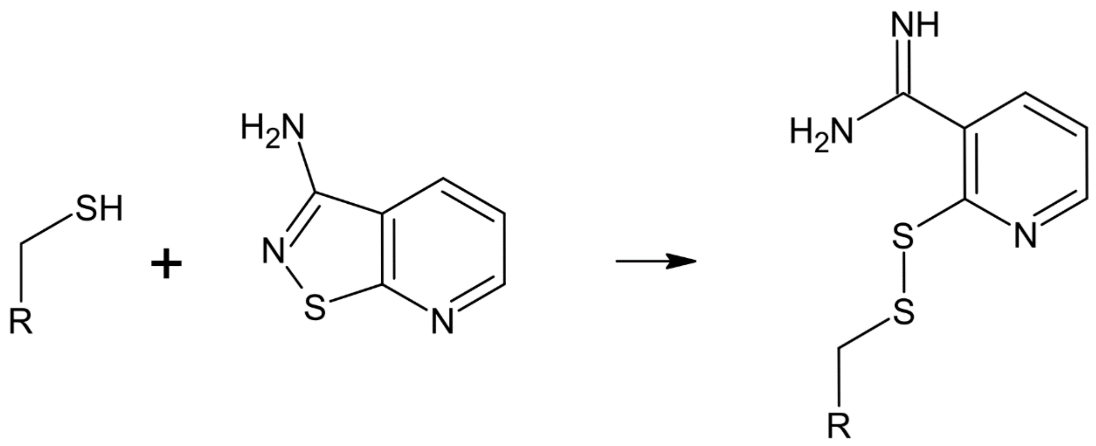

Supplementary Figure 9:
Scheme of covalent binding of fragment A09 to Cys126 resulting in ring-opening of the isothiazol.

Supplementary Table 1:

Average data collection and refinement statistics for screening campaigns

Screening	RT1	RT2	cryo1	cryo2	RT_single
**Data collection**					
Number of datasets	118	143	291	272	29
Patterns/dataset	33816 ± 5089	35761 ± 1576			
Hits/dataset	21669 ± 5914	25624 ± 7177			
Indexed patterns/ dataset	14045 ± 4990	16723 ± 5682			
Crystals/dataset	18973 ± 7335	24884 ± 9134	1	1	1
Space group	P 21 21 2	P 21 21 2	P 21 21 2	P 21 21 2	P 21 21 2
Cell dimensions					
a, *b*, *c* (Å)	70.8 ± 1.1, 91.4 ± 0.4, 45.3 ± 0.1	71.4 ± 0.6, 91.6 ± 0.2, 45.3 ± 0.1	68.1 ± 0.2, 89.8 ± 0.2, 44.9 ± 0.0	68.1 ± 0.2, 89.8 ± 0.2, 44.9 ± 0.1	69.3 ± 0.2, 90.8 ± 0.1, 45.2 ± 0.0
Resolution (Å) (low- high)	91.39 ± 0.35 - 1.43 ± 0.08	91.59 ± 0.21 - 1.48 ± 0.10	53.78 ± 2.28 - 1.22 ± 0.16	53.74 ± 2.13 - 1.41 ± 0.16	54.25 ± 3.21 - 1.88 ± 0.14
Resolution, high res shell (Å)	1.48 ± 0.09 - 1.43 ± 0.08	1.54 ± 0.10 - 1.48 ± 0.10	1.24 ± 0.16 - 1.22 ± 0.16	1.43 ± 0.17 - 1.41 ± 0.16	1.91 ± 0.15 - 1.88 ± 0.14
Resolution, based on I/σI=1 (SX data only)	1.45 ± 0.08	1.49 ± 0.08			
R_{split} or R_{merge} *	0.170 ± 0.048 (1.191 ± 0.402)	0.174 ± 0.048 (0.883 ± 0.239)	0.121 ± 0.141 (1.601 ± 0.729)	0.201 ± 0.110 (2.713 ± 0.454)	0.282 ± 0.107 (2.187 ± 0.392)
CC1/2	0.952 ± 0.031 (0.327 ± 0.124)	0.945 ± 0.074 (0.483 ± 0.141)	0.993 ± 0.041 (0.560 ± 0.094)	0.995 ± 0.019 (0.488 ± 0.076)	0.986 ± 0.015 (0.492 ± 0.119)
CC*	0.987 ± 0.008 (0.682 ± 0.122)	0.984 ± 0.039 (0.794 ± 0.094)	0.998 ± 0.014 (0.843 ± 0.045)	0.999 ± 0.005 (0.807 ± 0.042)	0.997 ± 0.004 (0.804 ± 0.066)
$I / \sigma I$	5.5 ± 1.2 (1.0 ± 0.3)	5.1 ± 1.0 (1.2 ± 0.4)	15.1 ± 4.4 (1.2 ± 0.2)	10.6 ± 2.0 (1.1 ± 0.1)	6.1 ± 1.8 (1.2 ± 0.2)
Completeness (%)	99.6 ± 0.8 (97.9 ± 5.2)	99.4 ± 5.5 (98.6 ± 11.7)	88.5 ± 7.3 (60.3 ± 27.3)	97.3 ± 4.1 (98.6 ± 3.8)	98.2 ± 2.2 (98.7 ± 1.5)
Redundancy	88.6 ± 39.7 (22.4 ± 9.5)	160.5 ± 59.9 (56.7 ± 26.3)	11.4 ± 1.7 (6.8 ± 2.7)	12.3 ± 1.8 (12.3 ± 1.9)	7.3 ± 1.2 (7.3 ± 1.4)
Wilson *B* ($Å^2$)	15.9 ± 1.0	18.2 ± 1.4	12.2 ± 1.8	12.9 ± 1.8	21.8 ± 1.9
**Refinement**					
Resolution (Å)	48.51 ± 4.66 - 1.43 ± 0.08	48.00 ± 4.30 - 1.48 ± 0.10	53.78 ± 2.28 - 1.22 ± 0.16	45.02 ± 1.47 - 1.41 ± 0.16	44.52 ± 7.36 - 1.88 ± 0.14
No. reflections	55687 ± 7951	50530 ± 8975	76253 ± 15445	56856 ± 19351	24179 ± 6208
R_{work} / R_{free}	0.160 ± 0.016/0.189 ± 0.017	0.166 ± 0.020/0.195 ± 0.022	0.148 ± 0.016/0.170 ± 0.022	0.161 ± 0.013/0.185 ± 0.016	0.181 ± 0.021/0.226 ± 0.022
No. atoms	2540.0 ± 21.6	2536.2 ± 25.3	2831.1 ± 38.5	2817.8 ± 28.0	2570.8 ± 21.3
Protein	2284.0 ± 0.0	2284.0 ± 0.0	2336.0 ± 0.0	2336.0 ± 0.0	2336.0 ± 0.0
Ligand/ion	2.0 ± 0.1	1.9 ± 0.3	6.1 ± 0.4	6.0 ± 0.2	6.0 ± 0.0
Water	254.0 ± 21.5	250.3 ± 25.3	489.0 ± 38.5	475.8 ± 28.0	228.8 ± 21.3
B-factors ($Å^2$)	21.4 ± 1.1	23.6 ± 1.3	16.4 ± 2.3	17.0 ± 2.1	28.4 ± 2.6
Protein	19.8 ± 1.1	22.1 ± 1.3	13.9 ± 2.6	14.6 ± 2.3	27.5 ± 2.8
Ligand/ion	11.9 ± 1.5	14.1 ± 1.5	28.4 ± 3.4	30.2 ± 3.0	47.9 ± 5.4
Water	35.1 ± 1.3	36.9 ± 1.7	28.0 ± 1.7	28.2 ± 1.8	37.5 ± 1.8
R.m.s. deviations					
Bond lengths (Å)	0.008 ± 0.003	0.009 ± 0.005	0.008 ± 0.003	0.006 ± 0.002	0.007 ± 0.005
Bond angles (°)	0.9 ± 0.2	1.0 ± 0.3	1.0 ± 0.2	0.9 ± 0.2	0.9 ± 0.4

*) Rsplit for serial crystal data, Rmerge for single crystal data

Values in parentheses are for highest-resolution shell.

Supplementary Table 2:

Data collection and refinement statistics for deposited structures

PDB ID	9G1A	9G1B	9G1C	9G1D	9G1E
compound	apo	A09	A12	E04	E07
**Data collection**					
Temperature (K)	296	296	296	296	296
Patterns/dataset	242923	31892	27740	35904	45451
Hits/dataset	103402	22800	19567	35699	22868
Indexed patterns/dataset	86850	19347	17836	20239	17415
Crystals/dataset	110307	26395	24381	28878	25150
Space group	P21212	P21212	P21212	P21212	P21212
Cell dimensions					
a, b, c (Å)	71.62, 91.69, 45.3	68.92, 90.67, 45.26	71.84, 91.69, 45.26	71.73, 91.65, 45.34	69.35, 90.88, 45.29
Resolution (Å) (low- high)	71.43 - 1.3	90.67 - 1.3	91.65 - 1.4	91.65 - 1.4	90.88 - 1.4
Resolution, high res shell (Å)	1.35 - 1.3	1.35 - 1.3	1.45 - 1.4	1.45 - 1.4	1.45 - 1.4
R_{split} or R_{merge} *	0.0837(1.1628)	0.1197(1.2702)	0.1053(1.2012)	0.1724(0.719)	0.1294(1.2606)
CC1/2	0.9893(0.432)	0.9584(0.223)	0.989(0.346)	0.9507(0.585)	0.967(0.271)
CC*	0.9973(0.7767)	0.9893(0.604)	0.9972(0.717)	0.9873(0.859)	0.9916(0.653)
$I / \sigma I$	9.1(0.9)	6.6(0.9)	6.7(0.8)	4.8(1.1)	6.5(0.9)
Completeness (%)	100(100)	99.91(99.21)	99.96(99.63)	100(100)	100(99.98)
Redundancy	349.1(49.4)	122.4(16.3)	127.6(20.7)	191.5(81.1)	132.1(31.3)
Wilson *B* (Å^2)	16.6	14.1	16.1	18.6	15.1
**Refinement**					
Resolution (Å)	45.85 - 1.3	54.87 - 1.3	45.85 - 1.4	56.49 - 1.4	45.44 - 1.4
No. reflections	73233	69781	59299	59367	56966
R_{work} / R_{free}	0.1383 / 0.1569	0.1405 / 0.1768	0.1522 / 0.1796	0.1736 / 0.1997	0.1472 / 0.182
No. atoms					
Protein	2308	2296	2326	2306	2395
Ligand/ion	2	16	11	14	15
Water	210	227	240	240	216
B-factors (Å^2)					
Protein	21.51	18.11	19.34	21.47	19.42
Ligand/ion	13.48	20.45	18.32	18.94	27.46
Water	35.45	32.92	35.36	35.9	33.74
R.m.s. deviations					
Bond lengths (Å)	0.005	0.018	0.006	0.011	0.006
Bond angles (°)	0.886	1.305	0.945	1.064	0.933
Ramachandran(%)					
outliers	0	0	0	0	0
allowed	0	0.74	0	0	0.74
favored	100	99.26	100	100	99.26
1-BDC (from pandda event map)	-	0.32	0.29	0.24	0.2
refined occupancy ligand	-	1	0.24	0.39	0.69
binding site	-	1	4	6	6

*) R_{split} for serial crystal data, R_{merge} for single crystal data

Values in parentheses are for highest-resolution shell.

PDB ID	9G1F	9G1G	9G1H	9G1I	9G1J
compound	E12	G08	H01	apo	A06
**Data collection**					
Temperature (K)	296	296	296	100	100
Patterns/dataset	45451	33265	31892		
Hits/dataset	19125	22118	22824		
Indexed patterns/dataset	9002	12787	17761		
Crystals/dataset	11419	16716	23860	3	1
Space group	P21212	P21212	P21212	P21212	P21212
Cell dimensions					
a, b, c (Å)	69.46, 90.92, 45.26	69.42, 91.03, 45.28	71.48, 91.59, 45.21	68.05, 89.81, 44.94	68.08, 89.92, 44.89
Resolution (Å) (low- high)	90.92 - 1.4	91.03 - 1.4	91.59 - 1.4	54.24 - 1.1	54.28 - 1.28
Resolution, high res shell (Å)	1.45 - 1.4	1.45 - 1.4	1.45 - 1.4	1.11 - 1.1	1.3 - 1.28
R_{split} or R_{merge} *	0.1848(2.018)	0.1123(2.6061)	0.1426(0.8043)	0.064(0.587)	0.163(2.837)
CC1/2	0.9589(0.15)	0.9879(0.128)	0.9671(0.512)	1(0.653)	0.994(0.499)
CC*	0.9895(0.511)	0.9969(0.476)	0.9916(0.823)	1(0.889)	0.998(0.816)
$I / \sigma I$	4.5(0.1)	6.6(0.7)	6.2(1.3)	30.4(1.1)	10.2(1.1)
Completeness (%)	99.29(94.7)	97.22(77.18)	100(100)	91.9(25.9)	99.6(99.6)
Redundancy	68.7(11.5)	127.8(4.9)	101.2(29.5)	30.8(2.3)	9.4(8.3)
Wilson *B* (Å^2)	14.7	14.4	15.4	10.5	12.3
**Refinement**					
Resolution (Å)	45.46 - 1.4	45.51 - 1.4	56.35 - 1.4	54.24 - 1.1	54.28 - 1.28
No. reflections	55813	54562	59188	104553	71212
R_{work} / R_{free}	0.1769 / 0.1995	0.1521 / 0.1901	0.1484 / 0.1678	0.1357 / 0.1482	0.1496 / 0.1748
No. atoms					
Protein	2396	2377	2293	2345	2348
Ligand/ion	14	17	19	6	14
Water	215	216	232	479	480
B-factors (Å^2)					
Protein	19.28	19.57	19.53	12.34	13.58
Ligand/ion	27.36	24.13	57.54	22.2	23.5
Water	33.64	34.44	32.7	25.54	27.09
R.m.s. deviations					
Bond lengths (Å)	0.005	0.006	0.005	0.007	0.005
Bond angles (°)	0.828	0.891	0.883	1.008	0.882
Ramachandran(%)					
outliers	0	0	0	0	0
allowed	0.74	0.37	0	1.11	0.37
favored	99.26	99.63	100	98.89	99.63
1-BDC (from pandda event map)	0.25	0.15	0.22	-	0.15
refined occupancy ligand	0.73	0.39	0.82	-	0.34
binding site	6	2	6	-	4

*) R_{split} for serial crystal data, R_{merge} for single crystal data
Values in parentheses are for highest-resolution shell.

PDB ID	9G1K	9G1L	9G1M	9RPX	9RPY
compound	A09	A12	B02	B06	B07
**Data collection**					
Temperature (K)	100	100	100	100	100
Patterns/dataset					
Hits/dataset					
Indexed patterns/dataset					
Crystals/dataset	1	1	1	1	1
Space group	P21212	P21212	P21212	P21212	P21212
Cell dimensions					
a, b, c (Å)	67.73, 89.61, 44.93	67.99, 89.79, 44.93	68.15, 89.79, 45	68.04, 89.91, 44.84	68.03, 89.75, 44.92
Resolution (Å) (low- high)	54.03 - 1.14	54.2 - 1.27	54.29 - 1.18	54.25 - 1.27	54.21 - 1.16
Resolution, high res shell (Å)	1.16 - 1.14	1.29 - 1.27	1.2 - 1.18	1.29 - 1.27	1.18 - 1.16
R_{split} or R_{merge} *	0.06(1.046)	0.137(1.988)	0.134(1.773)	0.116(1.591)	0.073(1.334)
CC1/2	1(0.562)	0.998(0.552)	0.997(0.41)	0.998(0.527)	1(0.549)
CC*	1(0.848)	0.999(0.843)	0.999(0.763)	0.999(0.831)	1(0.842)
$I / \sigma I$	20.3(1.2)	9.6(1)	10.8(1.1)	12.2(1.2)	16.7(1.2)
Completeness (%)	89.7(42.2)	96.1(80.2)	88.7(47.8)	92.3(99.3)	88.2(42.6)
Redundancy	11.9(4.9)	11.7(10.1)	12.7(7.7)	11.7(8.6)	12.6(7)
Wilson *B* ($Å^2$)	12.3	12.8	11.7	12.0	12.5
**Refinement**					
Resolution (Å)	54.03 - 1.14	54.2 - 1.27	54.29 - 1.18	54.25 - 1.27	54.21 - 1.16
No. reflections	89899	71270	81524	68229	84123
R_{work} / R_{free}	0.1403 / 0.1609	0.1459 / 0.1727	0.1413 / 0.1654	0.1489 / 0.179	0.1411 / 0.162
No. atoms					
Protein	2306	2341	2323	2340	2331
Ligand/ion	20	15	16	22	25
Water	460	461	464	490	455
B-factors ($Å^2$)					
Protein	14.16	14.92	13.44	13.06	14.77
Ligand/ion	22.16	23.58	25.46	25.98	23.87
Water	27.54	28.26	27.01	28.46	28.77
R.m.s. deviations					
Bond lengths (Å)	0.006	0.005	0.008	0.006	0.005
Bond angles (°)	1.033	0.915	1.055	0.98	0.895
Ramachandran(%)					
outliers	0	0	0	0	0
allowed	0.37	0.37	0.74	0.74	0.74
favored	99.63	99.63	99.26	99.26	99.26
1-BDC (from pandda event map)	0.37	0.16	0.12	0.18	0.12
refined occupancy ligand	1	0.42	0.33	0.41	0.26
binding site	1	4	4	6	5

*) R_{split} for serial crystal data, R_{merge} for single crystal data
Values in parentheses are for highest-resolution shell.

PDB ID	9RPZ	9RQ0	9RQ1	9RQ2	9RQ3
compound	B10	C06	C08	C09	C10
**Data collection**					
Temperature (K)	100	100	100	100	100
Patterns/dataset					
Hits/dataset					
Indexed patterns/dataset					
Crystals/dataset	1	1	1	1	1
Space group	P21212	P21212	P21212	P21212	P21212
Cell dimensions					
a, b, c (Å)	67.9, 89.69, 44.92	67.65, 89.57, 44.84	68.06, 89.74, 44.93	68.07, 89.79, 44.93	68.28, 89.8, 44.97
Resolution (Å) (low- high)	54.14 - 1.11	53.99 - 1.22	54.23 - 1.13	54.25 - 1.16	54.35 - 1.22
Resolution, high res shell (Å)	1.13 - 1.11	1.24 - 1.22	1.15 - 1.13	1.18 - 1.16	1.24 - 1.22
R_{split} or R_{merge} *	0.054(0.8)	0.1(1.87)	0.064(1.072)	0.073(1.208)	0.078(1.83)
CC1/2	1(0.637)	0.999(0.455)	0.999(0.463)	0.999(0.515)	1(0.446)
CC*	1(0.882)	1(0.791)	1(0.796)	1(0.825)	1(0.785)
$I / \sigma I$	21.9(1.3)	14.5(1.1)	19(1.2)	15.7(1.2)	15.4(1)
Completeness (%)	80.6(17.2)	92.1(73.5)	85.7(29.6)	87.8(41.1)	89.8(49.8)
Redundancy	12.1(4.2)	12.5(8.1)	11.9(4.8)	12.2(6.2)	12.4(9.5)
Wilson *B* (Å^2)	11.5	11.8	12.5	12.47	14.01
**Refinement**					
Resolution (Å)	54.14 - 1.11	53.98 - 1.22	54.23 - 1.13	54.24 - 1.16	54.35 - 1.22
No. reflections	87776	75116	89317	85084	74658
R_{work} / R_{free}	0.1312 / 0.15	0.1545 / 0.18	0.1383 / 0.1585	0.1415 / 0.1596	0.1451 / 0.1715
No. atoms					
Protein	2340	2347	2356	2347	2349
Ligand/ion	19	22	22	22	22
Water	479	467	468	466	452
B-factors (Å^2)					
Protein	13.18	14.04	14.07	14.3	16.21
Ligand/ion	22.51	24.28	24.42	22.52	26.62
Water	27.12	28.06	27.33	28	29.37
R.m.s. deviations					
Bond lengths (Å)	0.006	0.004	0.007	0.005	0.005
Bond angles (°)	0.927	0.804	1.017	0.895	0.855
Ramachandran(%)					
outliers	0	0	0	0	0
allowed	0.74	0.74	0.74	0.74	0.74
favored	99.26	99.26	99.26	99.26	99.26
1-BDC (from pandda event map)	0.13	0.2	0.1	0.16	0.13
refined occupancy ligand	0.63	0.57	0.37	0.58	0.29
binding site	6	6	6	6	6

*) R_{split} for serial crystal data, R_{merge} for single crystal data

Values in parentheses are for highest-resolution shell.

PDB ID	9RQ4	9RQ5	9G1N	9G1O	9RQ6
compound	D06	D07	E04	E07	E09
**Data collection**					
Temperature (K)	100	100	100	100	100
Patterns/dataset					
Hits/dataset					
Indexed patterns/dataset					
Crystals/dataset	1	1	1	1	1
Space group	P21212	P21212	P21212	P21212	P21212
Cell dimensions					
a, b, c (Å)	68.07, 89.67, 44.93	68.13, 89.79, 44.9	67.79, 89.48, 44.89	68.27, 89.88, 44.99	67.91, 89.69, 44.88
Resolution (Å) (low- high)	54.22 - 1.18	54.27 - 1.12	54.03 - 1.2	54.36 - 1.14	54.14 - 1.12
Resolution, high res shell (Å)	1.2 - 1.18	1.14 - 1.12	1.22 - 1.2	1.16 - 1.14	1.14 - 1.12
R_{split} or R_{merge} *	0.073(1.474)	0.059(0.812)	0.09(1.508)	0.067(0.903)	0.07(0.827)
CC1/2	1(0.564)	1(0.551)	0.999(0.576)	0.999(0.593)	0.999(0.611)
CC*	1(0.849)	1(0.843)	1(0.855)	1(0.863)	1(0.871)
$I / \sigma I$	16.7(1.1)	20.9(1.3)	15(1.1)	17.5(1.2)	17.7(1.3)
Completeness (%)	91.5(48.8)	84.7(28.6)	94(61.2)	92(50.5)	85.2(33.9)
Redundancy	12.1(7.3)	12(3.8)	12.5(8.2)	11.4(4.2)	11.8(4)
Wilson *B* ($Å^2$)	13.02	11.18	11.94	11.5	11.27
**Refinement**					
Resolution (Å)	54.22 - 1.18	54.27 - 1.12	54.03 - 1.2	54.36 - 1.14	54.14 - 1.12
No. reflections	84033	90640	80096	92667	89495
R_{work} / R_{free}	0.1394 / 0.163	0.134 / 0.1541	0.1408 / 0.1667	0.1388 / 0.1599	0.1374 / 0.1591
No. atoms					
Protein	2340	2356	2336	2352	2349
Ligand/ion	25	28	30	19	25
Water	465	472	472	465	480
B-factors ($Å^2$)					
Protein	14.81	12.73	12.66	13.41	12.57
Ligand/ion	25.55	19.12	21.64	20.25	22.35
Water	28.57	26.08	27	26.31	26.18
R.m.s. deviations					
Bond lengths (Å)	0.007	0.007	0.005	0.009	0.008
Bond angles (°)	0.998	0.993	0.958	1.072	1.028
Ramachandran(%)					
outliers	0	0	0	0	0
allowed	0.74	0.74	0.37	1.11	0.74
favored	99.26	99.26	99.63	98.89	99.26
1-BDC (from pandda event map)	0.14	0.22	0.23/0.17	0.25	0.11
refined occupancy ligand	0.38	0.61	0.71/0.68	1	0.37
binding site	6	6	5+6	6	6

*) R_{split} for serial crystal data, R_{merge} for single crystal data
Values in parentheses are for highest-resolution shell.

PDB ID	9G1P	9RQ7	9RQ8	9RQ9	9RQA
compound	E12	F02	F04	F08	F09
**Data collection**					
Temperature (K)	100	100	100	100	100
Patterns/dataset					
Hits/dataset					
Indexed patterns/dataset					
Crystals/dataset	1	1	1	1	1
Space group	P21212	P21212	P21212	P21212	P21212
Cell dimensions					
a, b, c (Å)	68.08, 89.75, 44.92	68.01, 90.03, 44.97	68.18, 89.59, 44.94	68.12, 89.72, 44.96	68.12, 90.14, 44.83
Resolution (Å) (low- high)	54.24 - 1.14	54.26 - 1.18	54.26 - 1.16	54.26 - 1.15	54.34 - 1.16
Resolution, high res shell (Å)	1.16 - 1.14	1.2 - 1.18	1.18 - 1.16	1.17 - 1.15	1.18 - 1.16
R_{split} or R_{merge} *	0.081(1.186)	0.074(1.243)	0.064(0.992)	0.068(1.039)	0.078(1.418)
CC1/2	0.999(0.528)	1(0.563)	0.999(0.518)	1(0.629)	1(0.492)
CC*	1(0.831)	1(0.849)	1(0.826)	1(0.879)	1(0.812)
I / σI	15.2(1.2)	16.9(1.1)	17.4(1.2)	17.3(1.1)	16.5(1.2)
Completeness (%)	85.6(31.2)	98.2(79.6)	89.9(49.4)	89.1(46.6)	88.7(44.1)
Redundancy	12.4(6.2)	11.6(4.7)	11.1(4.3)	11.9(5)	12.4(6.9)
Wilson *B* ($Å^2$)	11.51	12.28	12.88	12.08	12.14
**Refinement**					
Resolution (Å)	54.24 - 1.14	54.26 - 1.18	54.26 - 1.16	54.26 - 1.15	54.34 - 1.16
No. reflections	87308	89715	86584	88418	84473
R_{work} / R_{free}	0.1379 / 0.1637	0.1399 / 0.1636	0.1443 / 0.1681	0.1429 / 0.1631	0.1395 / 0.1638
No. atoms					
Protein	2345	2340	2327	2331	2306
Ligand/ion	22	38	26	20	23
Water	441	469	465	473	438
B-factors ($Å^2$)					
Protein	13.06	14.62	14.47	14.02	14.34
Ligand/ion	19.73	24.87	27.79	24.1	23.96
Water	25.76	28.11	28.34	27.55	28.32
R.m.s. deviations					
Bond lengths (Å)	0.009	0.007	0.006	0.007	0.009
Bond angles (°)	1.131	1	0.889	0.971	1.093
Ramachandran(%)					
outliers	0	0	0	0	0
allowed	1.11	0.74	0.37	0.74	0.74
favored	98.89	99.26	99.63	99.26	99.26
1-BDC (from pandda event map)	0.27	0.14/0.12	0.32	0.15	0.23
refined occupancy ligand	1	0.38/0.29	1	0.51	1
binding site	6	5+6	6	6	6

*) R_{split} for serial crystal data, R_{merge} for single crystal data
Values in parentheses are for highest-resolution shell.

PDB ID	9RQB	9RQC	9RQD	9G1Q	9RQE
compound	F10	G02	G03	G08	G11
**Data collection**					
Temperature (K)	100	100	100	100	100
Patterns/dataset					
Hits/dataset					
Indexed patterns/dataset					
Crystals/dataset	1	1	1	1	1
Space group	P21212	P21212	P21212	P21212	P21212
Cell dimensions					
a, b, c (Å)	68.01, 89.69, 44.94	67.73, 89.5, 44.91	67.83, 89.77, 44.87	68.07, 89.84, 44.94	68.06, 89.72, 44.95
Resolution (Å) (low- high)	54.19 - 1.27	54.01 - 1.15	54.12 - 1.17	54.25 - 1.11	54.23 - 1.16
Resolution, high res shell (Å)	1.29 - 1.27	1.17 - 1.15	1.19 - 1.17	1.13 - 1.11	1.18 - 1.16
R_{split} or R_{merge} *	0.123(2.363)	0.084(1.399)	0.087(1.37)	0.052(0.691)	0.08(1.484)
CC1/2	0.999(0.565)	0.999(0.594)	0.999(0.57)	1(0.633)	0.999(0.481)
CC*	1(0.85)	1(0.863)	1(0.852)	1(0.88)	1(0.806)
I / σI	11.8(1)	14(1.2)	14.2(1.1)	22.4(1.2)	16.5(1.2)
Completeness (%)	96.7(89.4)	86.4(39.4)	78.9(47)	88.7(25.7)	84.9(34.4)
Redundancy	13.1(11.2)	11.5(6)	12(6.5)	11.1(2.7)	13.1(8.8)
Wilson *B* ($Å^2$)	12.48	12.12	11.95	11.28	12.43
**Refinement**					
Resolution (Å)	54.19 - 1.27	54.01 - 1.15	54.12 - 1.17	54.25 - 1.11	54.22 - 1.16
No. reflections	70671	83356	74391	97107	81200
R_{work} / R_{free}	0.1471 / 0.1763	0.1434 / 0.1694	0.1434 / 0.1706	0.1332 / 0.1503	0.1411 / 0.1631
No. atoms					
Protein	2331	2331	2324	2336	2329
Ligand/ion	25	24	34	21	22
Water	439	464	458	494	448
B-factors ($Å^2$)					
Protein	15.24	13.86	13.7	12.8	14.28
Ligand/ion	24.29	22.82	22.36	21.11	22.12
Water	28.98	26.75	27.47	26.01	27.61
R.m.s. deviations					
Bond lengths (Å)	0.009	0.006	0.009	0.007	0.005
Bond angles (°)	1.105	0.958	1.123	1.02	0.882
Ramachandran(%)					
outliers	0	0	0	0	0
allowed	1.11	0.37	1.11	1.11	0.74
favored	98.89	99.63	98.89	98.89	99.26
1-BDC (from pandda event map)	0.21	0.19	0.22/0.16	0.13	0.15
refined occupancy ligand	0.55	0.63	0.65/0.4	0.5	0.51
binding site	6	6	5+6	2	6

*) R_{split} for serial crystal data, R_{merge} for single crystal data
Values in parentheses are for highest-resolution shell.

PDB ID	9G1R	9G1S	9RQF	9RQG	9RQH
compound	G12	H01	H06	H07	H12
**Data collection**					
Temperature (K)	100	100	100	100	100
Patterns/dataset					
Hits/dataset					
Indexed patterns/dataset					
Crystals/dataset	1	1	1	1	1
Space group	P21212	P21212	P21212	P21212	P21212
Cell dimensions					
a, b, c (Å)	67.79, 89.62, 44.89	67.9, 89.6, 44.91	68.05, 89.88, 44.95	68.17, 89.9, 44.95	67.96, 89.65, 44.99
Resolution (Å) (low- high)	54.07 - 1.24	54.11 - 1.12	54.25 - 1.13	54.32 - 1.16	54.16 - 1.15
Resolution, high res shell (Å)	1.26 - 1.24	1.14 - 1.12	1.15 - 1.13	1.18 - 1.16	1.17 - 1.15
R_{split} or R_{merge} *	0.083(1.294)	0.06(0.983)	0.053(0.821)	0.067(0.912)	0.089(1.159)
CC1/2	0.999(0.679)	1(0.532)	1(0.678)	0.999(0.69)	0.999(0.525)
CC*	1(0.899)	1(0.833)	1(0.899)	1(0.904)	1(0.83)
$I / \sigma I$	14.3(1.2)	18.6(1.2)	20.6(1.3)	17.3(1.2)	14.2(1.2)
Completeness (%)	95.2(97.8)	75.4(27.6)	86.5(41.7)	91.8(54.8)	87.3(41.2)
Redundancy	12.6(7.2)	11.9(4.7)	11.4(4.1)	11.9(4.9)	11.9(5.7)
Wilson *B* ($Å^2$)	13.31	12.38	12.41	11.9	11.33
**Refinement**					
Resolution (Å)	54.07 - 1.24	54.11 - 1.12	54.25 - 1.13	54.32 - 1.16	54.16 - 1.15
No. reflections	75092	79011	89056	89503	86175
R_{work} / R_{free}	0.1397 / 0.1647	0.1376 / 0.1604	0.1376 / 0.1572	0.1418 / 0.1641	0.139 / 0.1603
No. atoms					
Protein	2347	2311	2324	2331	2351
Ligand/ion	32	23	38	26	42
Water	448	440	451	453	449
B-factors ($Å^2$)					
Protein	14.87	13.69	13.98	13.77	13.12
Ligand/ion	25.8	30.77	23.55	23.54	22.16
Water	29.08	26.56	27.19	27.05	26.43
R.m.s. deviations					
Bond lengths (Å)	0.014	0.012	0.008	0.009	0.01
Bond angles (°)	1.305	1.254	1.085	1.06	1.143
Ramachandran(%)					
outliers	0	0	0	0	0
allowed	0.74	0.74	0.74	1.11	0.74
favored	99.26	99.26	99.26	98.89	99.26
1-BDC (from pandda event map)	0.19/0.19	0.2	0.14/0.12	0.1	0.17/0.17
refined occupancy ligand	0.57/0.61	0.63	0.28/0.16	0.24	0.37/0.37
binding site	3+7	6	5+6	6	5

*) R_{split} for serial crystal data, R_{merge} for single crystal data
Values in parentheses are for highest-resolution shell.

Supplementary Notes 1

Description of the binding mode of fragments found at sites 1-4

A09. The isothiazol ring of fragment A09 is opened due to the nucleophilicity of the thiolate group of Cys126 resulting in a covalent disulfide adduct of the compound as previously mechanistically described (*45*) (Supplementary Fig. 9). As a result of the isothiazol ring opening, a diamine moiety with delocalized positive charge is formed and additionally allows cation-π stacking with the Tyr131 sidechain (Fig. 5a-d). The fragment is stabilized by a strong hydrogen bond between one of the two amine groups and the backbone carbonyl of Gln129. The orientation of the pyridine ring is further stabilized by a hydrophobic contact with Gln129 Cβ.

A12. Fragment A12, binds non-covalently in the active site II (binding site 4) via three hydrogen bonds and coordinates the central Mn^{2+}-ion at site 4 (Fig. 4a and Fig. 5e-h). One of the two nitrogen heteroatoms of the oxadiazol ring forms a hydrogen bond with the hydroxyl group of Tyr39 (chain A), while the other nitrogen forms a hydrogen bond with the hydroxyl group of Tyr65 (chain B). A third hydrogen bond is formed between the hydroxylate and Thr9 Oγ. This hydroxylate substituent further coordinates the central manganese ion.

G08. Fragment G08 bind at site 2 not directly in the active site but on the bottom of the homodimer (Fig. 4a-c). One hydrogen bond is formed between the backbone carbonyl oxygen of Asp44 and the amine nitrogen of compound G08 (Fig. 5i-l). A second hydrogen bond is formed between the carbonyl oxygen of G08 and the backbone amide nitrogen of Leu119. Additionally, a hydrophobic interaction of the fragment with Ser118 is observed.

A06. Fragment A06 binds similar to fragment A12 to the open active site II (binding site 4). The three nitrogen atoms of the triazine ring form hydrogen bonds to the hydroxyl groups of the side chains of Thr9, Tyr39 and Tyr65 (Fig. 6a-d). There is further hydrophobic interaction with Cys48, which is located approximately perpendicular to the triazine ring plane.

B02. Also fragment B02 bind to active site II (binding site 4) via Mn^{2+} coordination and an additional hydrogen bond (Fig. 6e-h). The free electron pair of the nitrogen heteroatom in the isoquinoline ring is coordinating the Mn^{2+} ion (distance 2.5 Å). The fragment is further stabilized by a hydrogen bond with the hydroxyl group of Tyr65.

G12. Fragment G12 binds to site 3 which is, similar to fragment G08, on the bottom of the FosAKP homodimer (Fig. 4b and c). Here one hydrogen bond is formed between the carboxamide nitrogen of the fragment and the backbone carbonyl oxygen of Asp44 in chain A (Fig. 6i-l). A second hydrogen bond is formed between the carboxamide oxygen and the backbone amide nitrogen of Leu119 of chain B of the FosAKP homodimer. Further, the thiophen ring of G12 is stabilized by hydrophobic interaction with Ser41 and π-electron stacking with the indole moiety of His31 (chain A).

AlphaFold3 predictions compared with experimental cryo and RT structures

AlphaFold3 allows highly accurate prediction of protein/ligand complexes (*3*). We used the publicly available online server that allows prediction of proteins in complex with selected ions and ligands. This provided predictions of the structure of FosAKP in complex with Mn^{2+} and K^{+}. The top five structures were all predicted with high to very high confidence (Supplementary Fig. 8a). Comparison with our apo and existing crystal structures show that the AlphaFold3 models most closely resemble the Fosfomycin-bound structure of FosAKP (Supplementary Fig. 8b). A closer look at the active site reveals that the AlphaFold3 models are not able to correctly predict the apo conformation of the K^{+}-binding loop region. In particular, large differences are visible between the RT apo structure and AlphaFold3 predictions (Supplementary Fig. 8c).
