# Supplementary material for: Room-temperature X-ray fragment screening with serial crystallography
Source: Nat Commun. 2025 Oct 13;16:9089. doi: 10.1038/s41467-025-64918-6 (PMC12518807; doi:10.1038/s41467-025-64918-6)
Supplement: Supplementary file 2 — Description of Additional Supplementary Files [file 41467_2025_64918_MOESM2_ESM.docx]

# Description of additional supplementary data

## Supplementary Data 1:

F2X fragment library. List of fragments in the F2X entry screen as used in this study, including vendor, order and lot numbers.
