# Supplementary material for: Room-temperature X-ray fragment screening with serial crystallography
Source: Nat Commun. 2025 Oct 13;16:9089. doi: 10.1038/s41467-025-64918-6 (PMC12518807; doi:10.1038/s41467-025-64918-6)
Supplement: Supplementary file 4 — Reporting Summary [file 41467_2025_64918_MOESM4_ESM.pdf]

nature portfolio

Corresponding author(s): Alke Meents
Sebastian Guenther

Last updated by author(s): Sep 11, 2025

Reporting Summary

Nature Portfolio wishes to improve the reproducibility of the work that we publish. This form provides structure for consistency and transparency in reporting. For further information on Nature Portfolio policies, see our Editorial Policies and the Editorial Policy Checklist.

Statistics

For all statistical analyses, confirm that the following items are present in the figure legend, table legend, main text, or Methods section.

n/a	Confirmed	
☐	☒	The exact sample size (*n*) for each experimental group/condition, given as a discrete number and unit of measurement
☒	☐	A statement on whether measurements were taken from distinct samples or whether the same sample was measured repeatedly
☒	☐	The statistical test(s) used AND whether they are one- or two-sided<br>*Only common tests should be described solely by name; describe more complex techniques in the Methods section.*
☒	☐	A description of all covariates tested
☒	☐	A description of any assumptions or corrections, such as tests of normality and adjustment for multiple comparisons
☐	☒	A full description of the statistical parameters including central tendency (e.g. means) or other basic estimates (e.g. regression coefficient) AND variation (e.g. standard deviation) or associated estimates of uncertainty (e.g. confidence intervals)
☒	☐	For null hypothesis testing, the test statistic (e.g. *F*, *t*, *r*) with confidence intervals, effect sizes, degrees of freedom and *P* value noted<br>*Give P values as exact values whenever suitable.*
☒	☐	For Bayesian analysis, information on the choice of priors and Markov chain Monte Carlo settings
☒	☐	For hierarchical and complex designs, identification of the appropriate level for tests and full reporting of outcomes
☒	☐	Estimates of effect sizes (e.g. Cohen's *d*, Pearson's *r*), indicating how they were calculated

Our web collection on statistics for biologists contains articles on many of the points above.

Software and code

Policy information about availability of computer code

Data collection	For data collection, Janus, an in-house developed beamline control software was used (no version number available yet).
Data analysis	autoproc (1.0.5), XDS (Jan 10, 2022 (BUILT 20220820), CrystFEL (0.10.1), cluster4x (within Vagabond 0.3.30), PanDDA (0.2.14), ccp4 (7.0.055), phenix (1.20 and 1.21.2-5419), Origin (2022b/9.9.5.167), Xtrapol8 (1.2.9), PyMOL (3.1.0a0), PoseEdit (accessed through the server at https://proteins.plus)

For manuscripts utilizing custom algorithms or software that are central to the research but not yet described in published literature, software must be made available to editors and reviewers. We strongly encourage code deposition in a community repository (e.g. GitHub). See the Nature Portfolio guidelines for submitting code & software for further information.

Data

Policy information about availability of data

All manuscripts must include a data availability statement. This statement should provide the following information, where applicable:

- Accession codes, unique identifiers, or web links for publicly available datasets
- A description of any restrictions on data availability
- For clinical datasets or third party data, please ensure that the statement adheres to our policy

All structure models and structure factors, including PanDDA event maps, have been deposited in the PDB with the IDs 9G1A [https://doi.org/10.2210/pdb9G1A/pdb], 9G1B [https://doi.org/10.2210/pdb9G1B/pdb], 9G1C [https://doi.org/10.2210/pdb9G1C/pdb], 9G1D [https://doi.org/10.2210/pdb9G1D/pdb], 9G1E [https://doi.org/10.2210/pdb9G1E/pdb], 9G1F [https://doi.org/10.2210/pdb9G1F/pdb], 9G1G [https://doi.org/10.2210/pdb9G1G/pdb], 9G1H [https://doi.org/10.2210/

pdb9G1H/pdb], 9G1I [https://doi.org/10.2210/pdb9G1I/pdb], 9G1J [https://doi.org/10.2210/pdb9G1J/pdb], 9G1K [https://doi.org/10.2210/pdb9G1K/pdb], 9G1L [https://doi.org/10.2210/pdb9G1L/pdb], 9G1M [https://doi.org/10.2210/pdb9G1M/pdb], 9RPX [https://doi.org/10.2210/pdb9RPX/pdb], 9RPY [https://doi.org/10.2210/pdb9RPY/pdb], 9RPZ [https://doi.org/10.2210/pdb9RPZ/pdb], 9RQ0 [https://doi.org/10.2210/pdb9RQ0/pdb], 9RQ1 [https://doi.org/10.2210/pdb9RQ1/pdb], 9RQ2 [https://doi.org/10.2210/pdb9RQ2/pdb], 9RQ3 [https://doi.org/10.2210/pdb9RQ3/pdb], 9RQ4 [https://doi.org/10.2210/pdb9RQ4/pdb], 9RQ5 [https://doi.org/10.2210/pdb9RQ5/pdb], 9G1N [https://doi.org/10.2210/pdb9G1N/pdb], 9G1O [https://doi.org/10.2210/pdb9G1O/pdb], 9RQ6 [https://doi.org/10.2210/pdb9RQ6/pdb], 9G1P [https://doi.org/10.2210/pdb9G1P/pdb], 9RQ7 [https://doi.org/10.2210/pdb9RQ7/pdb], 9RQ8 [https://doi.org/10.2210/pdb9RQ8/pdb], 9RQ9 [https://doi.org/10.2210/pdb9RQ9/pdb], 9RQA [https://doi.org/10.2210/pdb9RQA/pdb], 9RQB [https://doi.org/10.2210/pdb9RQB/pdb], 9RQC [https://doi.org/10.2210/pdb9RQC/pdb], 9RQD [https://doi.org/10.2210/pdb9RQD/pdb], 9G1Q [https://doi.org/10.2210/pdb9G1Q/pdb], 9RQE [https://doi.org/10.2210/pdb9RQE/pdb], 9G1R [https://doi.org/10.2210/pdb9G1R/pdb], 9G1S [https://doi.org/10.2210/pdb9G1S/pdb], 9RQF [https://doi.org/10.2210/pdb9RQF/pdb], 9RQG [https://doi.org/10.2210/pdb9RQG/pdb], 9RQH [https://doi.org/10.2210/pdb9RQH/pdb]. PDB 5V91 [https://doi.org/10.2210/pdb5V91/pdb] was initially used as starting model structure determination. All models and structure factor files derived from the automatic refinement pipeline and described in this manuscript are provided at https://doi.org/10.5281/zenodo.15863149 [https://doi.org/10.5281/zenodo.15863149]. The results from the PanDDA analysis used as starting points for the deposited structures and isomorphous difference and extrapolated structure factor maps for the RT hit fragments are provided there as well.
Source data for Fig. 2b/c, 3a and Supplementary Fig. 2c, 3a, 7s are provided with the paper.

Research involving human participants, their data, or biological material

Policy information about studies with human participants or human data. See also policy information about sex, gender (identity/presentation), and sexual orientation and race, ethnicity and racism.

Reporting on sex and gender	n/a
Reporting on race, ethnicity, or other socially relevant groupings	n/a
Population characteristics	n/a
Recruitment	n/a
Ethics oversight	n/a

Note that full information on the approval of the study protocol must also be provided in the manuscript.

Field-specific reporting

Please select the one below that is the best fit for your research. If you are not sure, read the appropriate sections before making your selection.

- [x] Life sciences
- [ ] Behavioural & social sciences
- [ ] Ecological, evolutionary & environmental sciences

For a reference copy of the document with all sections, see nature.com/documents/nr-reporting-summary-flat.pdf

Life sciences study design

All studies must disclose on these points even when the disclosure is negative.

Sample size	Sample sizes were not predetermined. For the screenings conducted with single crystal data collection, 2 to 3 crystals were prepared for each fragment to account for variability in sample quality to ensure that at least one dataset for each fragment would be available. For the datasets collected by serial crystallography, one compartment of a fixed-target sample holder was prepared with one fragment, each compartment containing several thousand crystals. The final dataset is generated from the still diffraction images collected from all these crystals (on average 19000 and 25000 for RT1 and RT2, respectively).
Data exclusions	No data was excluded for data analysis for datasets RT1, RT2, cryo1 and cryo2. For dataset cryo2, datasets (n=3) with a resolution worse than 3A were excluded from PanDDA analysis. For dataset RT_single, datasets (n=3) with an Rpim value > 0.2 after automatic data processing were excluded from analysis due to their overall bad quality.
Replication	Each of the screening was conducted twice. For the cryo datasets we collected data from two to three crystals per fragment. For the room-temperature datasets we collected for most fragments only one dataset, which each consisted of about 19000 (RT1) and 25000 (RT2) crystals. Replication of hit identification within the cryo and RT screens was not complete. Likly reasons include better diffraction quality (in particular cryo1 vs. cryo2) and different levels of heterogeneity with RT1 and RT2 that possibly led to failure to detect the same ligands in both RT screen.
Randomization	No randomization was conducted for this study as the samples within each screen were not allocated into experimental groups.
Blinding	For data collection, crystals for cryo screens were assigned IDs and researchers did not know which ligand was assigned to which crystal. For RT screens 12 ligands were grouped on a single fixed-target sample holder. Here no blinding was done. Clustering of data prior to hit finding with PanDDA was done based on raw diffraction data, independent of any ligand information.

Reporting for specific materials, systems and methods

We require information from authors about some types of materials, experimental systems and methods used in many studies. Here, indicate whether each material, system or method listed is relevant to your study. If you are not sure if a list item applies to your research, read the appropriate section before selecting a response.

Materials & experimental systems

n/a	Involved in the study
☒	☐ Antibodies
☒	☐ Eukaryotic cell lines
☒	☐ Palaeontology and archaeology
☒	☐ Animals and other organisms
☒	☐ Clinical data
☒	☐ Dual use research of concern
☒	☐ Plants

Methods

n/a	Involved in the study
☒	☐ ChIP-seq
☒	☐ Flow cytometry
☒	☐ MRI-based neuroimaging

Plants

Seed stocks	n/a
Novel plant genotypes	n/a
Authentication	n/a
